# “Giving something back”: A systematic review and ethical enquiry into public views on the use of patient data for research in the United Kingdom and the Republic of Ireland

**DOI:** 10.12688/wellcomeopenres.13531.2

**Published:** 2019-01-17

**Authors:** Jessica Stockdale, Jackie Cassell, Elizabeth Ford

**Affiliations:** 1 Department of Primary Care and Public Health, Brighton and Sussex Medical School, Brighton, UK; 2 Department of Philosophy, School of History, Art History and Philosophy, University of Sussex, Brighton, UK

**Keywords:** Privacy, Patient Data, Electronic Health Records, Governance, Public, Engagement, Ethics

## Abstract

**Background**: Use of patients’ medical data for secondary purposes such as health research, audit, and service planning is well established in the UK. However, the governance environment, as well as public understanding about this work, have lagged behind. We aimed to systematically review the literature on UK and Irish public views of patient data used in research, critically analysing such views though an established biomedical ethics framework, to draw out potential strategies for future good practice guidance and inform ethical and privacy debates.

**Methods**: We searched three databases using terms such as patient, public, opinion, and electronic health records. Empirical studies were eligible for inclusion if they surveyed healthcare users, patients or the public in UK and Ireland and examined attitudes, opinions or beliefs about the use of patient data for medical research. Results were synthesised into broad themes using a framework analysis.

**Results**: Out of 13,492 papers and reports screened, 20 papers or reports were eligible. While there was a widespread willingness to share patient data for research for the common good, this very rarely led to unqualified support. The public expressed two generalised concerns about the potential risks to their privacy. The first of these concerns related to a party’s competence in keeping data secure, while the second was associated with the motivation a party might have to use the data.

**Conclusions**: The public evaluates trustworthiness of research organisations by assessing their competence in data-handling and motivation for accessing the data. Public attitudes around data-sharing exemplified several principles which are also widely accepted in biomedical ethics. This provides a framework for understanding public attitudes, which should be considered in the development in any guidance for regulators and data custodians. We propose four salient questions which decision makers should address when evaluating proposals for the secondary use of data

## Introduction

The use of patients’ medical data for secondary purposes such as health research, audit, and service planning is well established in the UK, and technological innovation in analytical methods for new discoveries using these data resources is developing quickly. Data scientists have developed, and are improving, many ways to extract and process information in medical records. This continues to lead to an exciting range of health related discoveries, improving population health and saving lives. Nevertheless, as the development of analytic technologies accelerates, the decision-making and governance environment as well as public views and understanding about this work, has been lagging behind ^1^.

### Public opinion and data use

A range of small studies canvassing patient views, mainly in the USA, have found an overall positive orientation to the use of patient data for societal benefit ^2– 7^. However, recent case studies, like NHS England’s ill-fated Care.data scheme, indicate that certain schemes for secondary data use can prove unpopular in the UK. Launched in 2013, Care.data aimed to extract and upload the whole population’s general practice patient records to a central database for prevalence studies and service planning ^8^. Despite the stated intention of Care.data to “make major advances in quality and patient safety” ^8^, this programme was met with a widely reported public outcry leading to its suspension and eventual closure in 2016. Several factors may have been involved in this failure, from the poor public communication about the project, lack of social licence ^9^, or as pressure group MedConfidential suggests, dislike of selling data to profit-making companies ^10^. However, beyond these specific explanations for the project’s failure, what ignited public controversy was a concern with the impact that its aim to collect and share data on a large scale might have on patient privacy. The case of Care.data indicates a reluctance on behalf of the public to share their patient data, and it is still not wholly clear whether the public are willing to accept future attempts at extracting and linking large datasets of medical information. The picture of mixed opinion makes taking an evidence-based position, drawing on social consensus, difficult for legislators, regulators, and data custodians who may respond to personal or media generated perceptions of public views. However, despite differing results of studies canvassing public views, we hypothesise that there may be underlying ethical principles that could be extracted from the literature on public views, which may provide guidance to policy-makers for future data-sharing.

### Governance and legal framework of data use

Since 2018, the General Data Protection Regulation (GDPR) has governed the use of patients’ medical data in the EU and UK, superseding the Data Protection Act in the UK (1998). GDPR covers personal, or patient identifiable data in the UK, and defines pseudonymised data, which can be traced back to the individual using a study or database specific ID code, as personal data. Patient data can be used for direct care or audit and healthcare quality improvement projects without consent as these are seen as primary use of data. Research, however, is a secondary use of such data, as it is a use different from the originally declared purpose of data collection. Research organisations thus need a “legal basis” for processing the data even when identifiers are stripped, if individuals are still potentially re-identifiable by a pseudonymisation code ^11^. One such legal basis is individual consent for use of the data in this way, and a second is “a task in the public interest”. For research, such processing may be justified in terms of research for the public good, as long as appropriate safeguards are in place to reduce potential harms to the data subject and ensure respect for the principle of data minimisation.

Reducing potential harms to the data subject involves taking a range of precautions to reduce the risk that an individual patient could be re-identified. Removing the pseudonymisation code and aggregating the data to a level at which re-identification is not possible is the surest way of reducing such harm, but often renders the data less usable for research purposes and destroys the ability to link the data to other sources of health information. Research teams usually robustly protect patient data with computing security systems, which do not allow the data to be downloaded, or unapproved datasets to be uploaded and linked to the sensitive data. They also ensure only trusted and trained users are permitted access to the data. If data are to be released to the public, this is usually done only after data have been aggregated so that they have become truly anonymous.

### Striking a balance

While it is clearly important to make sure patient privacy is protected, it is also argued that the societal benefit of medical research using patient data, should be given ethical weight. This is argued on the basis that harms to patients may occur where these rich data sources are not used to improve our understanding of health conditions and treatments ^12^. While individual privacy and societal benefit are often portrayed as being in opposition, for the future of health data research, a way to achieve both to the satisfaction of patients, clinicians, legislators and researchers must be found. Recent work has sought to identify the key issues of patient views on data-sharing ^13^. However, few syntheses of patient views have additionally aimed to identify the implicit reasoning on which patients rely to justify the responses they give. Identifying a framework which describes the core moral or ethical values underlying public views may help us to predict the reaction of the public to new data sharing challenges in the future. Since the tension between data sharing and privacy is an ethical tension (to the extent that it involves what states of affairs *ought* to obtain, morally speaking), we are interested in the ethical dimensions of such patient reasoning. To this end, we propose to draw on core principles of biomedical ethics. First suggested in 1978 and 1979 in two forms, by Beauchamp and Childress for ethical conduct within medicine ^14^, and in the Belmont report ^15^ for ethical conduct in healthcare research. Core principles include the concepts of respect for autonomy, beneficence, non-maleficence, and justice. A similar set of principles were applied to information technology research in science and technology by Menlo in a 2012 report ^16^. We aimed to use these widely accepted principles as a tool to identify underlying themes expressed in patient views, and as a lens to discuss the findings. We also aimed to bring together these core principles with the newly adopted social licence theory for patient data research ^9^. This theory suggests that by voluntarily adhering to social codes of trustworthy and responsible behaviour that go beyond legal or regulatory frameworks, and by honouring additional safeguards, organisations can engender trust from the public for schemes which may initially be controversial.

In this study, we therefore aimed to systematically review and thematically analyse UK and Irish studies exploring patient and public views on patients’ medical data being used for the secondary purpose of research, and aimed to understand and map these views onto established biomedical ethical principles. We aimed to make suggestions for consideration by ethics committees and regulators to ensure that such research operates in a transparent and trustworthy way, with the aim of maximising the potential for the public to grant a social licence for such research to operate.

## Methods

We followed the PRISMA guidelines for the conduct and reporting of this review ^17^.

### Search strategy

We searched PubMed, Web of Science, and Scopus between 03/10/16 and 11/10/16 using the following search string: (Public OR Patient OR People) AND (Attitudes OR Knowledge OR Opinions OR Views OR Perceptions) AND ("Care.data" OR "Electronic Health Record" OR "Electronic Health Data" OR "Electronic Medical Record" OR "Electronic Medical Data" OR "Personal Health Information" OR "Personal Health Record" OR "Electronic Patient Information" OR "Electronic Patient Data" OR "Electronic Patient Record" OR "Data linkage" OR "Data sharing") AND (Research). We restricted our search to publications from 2006–2016 inclusive. We also searched the grey literature using the search string: "public attitudes" AND "sharing" AND "health data" on Google (in June 2017). The first 20 results were selected and screened. The following inclusion criteria were then applied:

1. Empirical studies using any methods reported as a full length peer review manuscript or published report.
2. Healthcare users, patients or the wider public as participants
3. Examining views, attitudes, opinions, perspectives, thoughts, awareness or acceptance about the topic of use of patient data for medical research.
  3a. Patient data for medical research includes electronic hospital records, electronic general practice records, and data extracted from these records, for example cancer registries and national disease databases (summarised as patient data or EHRs).
4. Studies using a UK or Irish sample, written in English. We chose to keep our review to these two countries because of similarities in their socialised healthcare systems, and because of the well-established use of patient data within these jurisdictions.

Studies were excluded if they were:

1. Focused more broadly on digital technologies in health care where the focus was on use of digital methods or records rather than public attitudes
2. Focused on patient and practitioner attitudes to analogous areas such as biorepositories, genetic testing and genomic research or personal data not exclusively related to health.
3. Non-empirical reviews of legislation, policy, ethical challenges etc.

Using these criteria, the articles extracted from the literature search were screened based on their title, then abstract (by author JS), then finally the choice of full text papers for the review was undertaken by two authors (JS and EF).

### Quality Assessment

Study quality was assessed using the Mixed Methods Appraisal Tool (MMAT) ^18^. This tool was designed for the appraisal of studies in mixed methods systematic reviews and attempts to appraise the quality of methodology, rather than the quality of reporting. All studies meeting the inclusion criteria above were assessed using six criteria. The first two criteria are the same for all studies: is there a clear research question or objective, and does the data collected address the research question or objective. A further four questions were specific to the study type. Studies were given a score out of six depending on how many of the six criteria they met, and were rejected if they did not meet at least the first two criteria. Two papers were excluded on the basis of scoring zero on all criteria.

While quantitative papers are generally seen as of higher quality, we believed that the in-depth exploration of human reasoning for participant perspectives and decisions, illustrated in the qualitative studies, could offer a greater understanding to underpin our moral and ethical interpretation. Thus we treated quantitative and qualitative studies as having equal value in the analysis if they met quality criteria. 

### Data extraction

We extracted author names, dates, location, type of study (qualitative or quantitative), methods used, number of participants, their backgrounds or roles, ages, genders, and the study findings which fitted into the themes relating to research questions reported below.

### Synthesis of results

The full text of eligible articles was read iteratively by two authors (JS and EF) with the aim of extracting coherent themes. In the first iteration of reading and coding the results of the papers, nine questions arose, which formed the basic direction of the inquiry.

1. Are patients/public aware of electronic health records (EHRs) and their secondary uses?
2. Are patients/public concerned about the privacy and security of their medical data?
3. Are patients/public willing to share their medical data for research, policy and planning?
4. What consent model do patients/public prefer?
5. How does data being identifiable or anonymised affect patient/public preferences?
6. Which organisations are most and least trusted with patients’/public data?
7. What are the reported perceptions of risks and benefits of sharing medical data for research?
8. Are there any other ways in which willingness to share could be increased?
9. Is there any differences between demographic groups concerned with the sharing of EHRs?

A framework ^19^ was created with a column for each of the nine questions and data was extracted from each study where it fitted into these categories. Following this data extraction, the two authors (EF and JS) discussed refining and combining extracted data into as smaller number of themes. In a second iteration of data extraction, authors re-read articles and extracted data into seven themes. For interpretation and synthesis, a data driven approach was taken, trying to make meaning from first order data reported in the papers (i.e. statistics or participant quotes). Where themes were populated mainly by summary of quantitative data, a straightforward report of papers’ findings is given.

Where contributing papers were mainly qualitative e.g. in the Trust theme, we undertook a deeper analysis directed by Braun and Clarke’s guidance on qualitative thematic analysis ^20^, an approach recommended for meta-synthesis by Dixon-Woods *et al.* ^21^ and Thomas and Harden ^22^. By taking an interpretive approach ^23^ to the synthesis of the data, we examined “the *underlying* ideas, assumptions, and conceptualisation – and ideologies – that are theorized as shaping or informing the semantic content of the data” (p.84) ^20^. This was shaped and directed by Beauchamp and Childress’ four principles of bioethics ^14^, which enabled us to gain a better understanding of the emerging moral meaning, and moral values conveyed by the study participants in these themes. As far as the authors are aware, there are no pre-existing applications of this framework to study patient attitudes. However, this general approach has been taken before using The Belmont Report to identify stakeholder views on technology-enabled research ^24^. While the four principles have drawn criticism elsewhere ^25, 26^ they continue to be extremely influential in evaluations of ethical dilemmas in health care, and a useful framework with which to identify moral values in participant decision-making.

## Results

A total of 13,472 peer-reviewed papers were found through the systematic search, as well as 20 reports found through the grey literature search. Of these, 20 UK and Ireland based papers met the inclusion criteria and were included in the review ^4, 27– 45^ ( Supplementary File 2). Studies which reported time periods indicated that data was collected from 2004 to 2016, although seven studies published between 2011 and 2016 did not report the data collection period. Research participants included patients, service-users, lay persons, those living with chronic conditions, and the general public ranging from 16 years of age to over 75. Five of the studies included the views of health researchers, health professionals, industry experts, NHS managers and other key stakeholders. Seven of the papers were quantitative, using surveys or structured questionnaires. Ten of the studies were qualitative, using focus groups and one-to-one interviews, and there were three mixed methods studies. Details of studies are reported in Table 1.

**Table 1.** Included study characteristics.

Study no.	Reference	Method	Setting	Data Collection Period	Sample	Quality Score
27	Audrey, S., *et al.,* BMC Medical Research Methodology, 2016. 16: p. 34	Qualitative: focus groups with participants from ALSPAC.	Bristol, England	Not stated	Total n=55, 56.4% female 43.6% male Ages: 17–19.	5
28	Baird, W., *et al.,* Journal of Medical Ethics, 2009. 35(2): p. 92–96.	Qualitative: focus groups and interviews.	England and Northern Ireland	February and July 2006	Total n=68 Focus groups: Patients with MS and stakeholders (n=55) Interviews: Health and social care professionals an academics (n=13)	5
29	Barrett, G., *et al.,* British Medical Journal, 2006. 332(7549): p. 1068–1070.	Quantitative: survey run by the Office of national statistics	England, Wales and Scotland	March and April 2005	Total n=2872 46% male 54% female Ages: 16–44 46%; 45–64 35%; 65+ 20%	6
30	Buckley, B.S., A.W. Murphy, and A.E. MacFarlane, Journal of Medical Ethics, 2011. 37(1): p. 50–55.	Quantitative: postal electoral roll-based questionnaire survey.	Republic of Ireland	Not stated	Total n=1575 27.6% male 71.6% female Ages: 18–75+	4
31	Campbell, B., *et al.,* Quality and Safety in Health Care, 2007. 16(6): p. 404–408.	Quantitative: postal questionnaire	South-West England	October to December 2004	Total n=166 patients recently discharged from the care of 78 bed-holding consultants across all specialties at the Royal Devon and Exeter Hospital.	5
32	CM Insight and Wellcome Trust, Summary report of qualitative research into public attitudes to personal data and linking personal data. 2013.	Qualitative: focus groups and one-to-one telephone interviews.	London, Midlands and Norfolk, England	29 April to 12 May 2013	Total n=50, Ages: 18–70 Focus group respondents were recruited as owners of store loyalty cards, smart phones and social media users. Telephone interviewees were recruited as especially pro-privacy or cautious about sharing personal data.	3
4	Clerkin, P., *et al.,* Family Practice, 2013. 30(1): p. 105–112.	Qualitative: focus groups.	West Republic of Ireland	Not stated	Total n=35, female (n=18) male (n=17) Ages: 18–35(n=2); 36–55(n=14); 56–70 (n=19).	6
33	Grant, A., *et al.*, BMC Health Services Research, 2013. 13: p. 422.	Qualitative: focus groups and semi-structured interviews.	Tayside and Lothian, Scotland	Between February and June 2011	Total n=64, Focus Groups: Patients (n=37), Health services researchers (n=10) Interviews: GPs and Practice managers (n=17)	5
34	Haddow, G., *et al.,* Journal of Evaluation in Clinical Practice, 2011. 17(6): p. 1140–1146.	Qualitative: focus groups.	North East Scotland	May and June 2009	Total n=19, female (n=12), male (n=6), Unstated (n=1) Ages: <60 (n=1); 60–74 (n=15); +75 (n=3).	5
35	Hays, R. and G. Daker-White, BMC Public Health, 2015. 15: p. 838.	Qualitative: using tweets.	Twitter	Over 18 days during February and March 2014	3537 tweets containing the hashtag #caredata; 904 contributors	6
36	Hill, E.M., *et al.,* BMC Medical Research Methodology, 2013. 13: p. 72.	Qualitative: focus groups.	England, Wales, Scotland and Northern Ireland	Not stated	Total n=19, 100% male Ages: 54–69; mean age 61.	4
37	Ipsos Mori, Medical Research Council, The use of personal health information in medical research general public consultation. 2007.	Mixed methods study. Qualitative: workshops and interviews Quantitative: face-to-face survey	England, Wales, Scotland and Northern Ireland	Quant: 14–18 Sept 2006; Qual 29/7- 5/8 2006	Quant: 2106 people aged 15+; Qual: Total n=69 Workshops: General public (n=63) Interviews: disabled people, and people with chronic illnesses/or their carers (n=6)	3
38	Ipsos Mori, Macmillan Cancer Support, Cancer Research UK, Perceptions of the Cancer Registry: Attitudes towards and awareness of cancer data collection. 2016.	Quantitative: online survey.	England	13 June to 4 July 2016	Total n=2,033 Adults who have, or have had cancer (PLWC) (n=1,033) Adults from the general public (n=1,000). All 18+	5
39	Ipsos Mori, Wellcome Trust, The one-way mirror: Public attitudes to commercial access to health data. 2016.	Mixed methods study. Quantitative: face-to-face survey Qualitative workshops	England, Wales, Scotland and Northern Ireland	September to December 2015	Quant: n = 2017, age 16+; Qual n= 247; Members of the general public, patients, and ALSPAC cohort members (n=212) GPs and hospital doctors (n=35)	3
40	Ipsos Mori, Wellcome Trust, Wellcome Trust monitor report wave 3: Tracking public views on science and biomedical research. 2016.	Quantitative: questionnaire using face-to-face Computer-Assisted Personal Interviewing (CAPI).	England, Wales, Scotland and Northern Ireland	2 June to 1 November 2015	Total n=1,524 Ages: 18+	5
41	Luchenski, S.A., *et al.,* Journal of Medical Internet Research, 2013. 15(8).	Quantitative: cross sectional, self-completed questionnaire survey.	West London, England	Six weeks from 1 August 2011	Total n=2857, 59.5% female, 40.5% male Ages: 18–75+	5
42	Papoutsi, C., *et al.* BMC Medical Informatics and Decision Making, 2015. 15(1): p. 124.	Mixed methods study. Quantitative: questionnaire survey Qualitative: focus groups and interviews.	West London, England	Between August 2011 and April 2013	Quant: n=2761, 59.1% female; Qual: n=160, Patients (n=114). Health professionals and researcher total not stated Interviews: Patients who did not wish to join group discussions (n=6).	4
43	Riordan, F., *et al.,* International Journal of Medical Informatics, 2015. 84(4): p. 237–247.	Quantitative: Crosssectional, self-completed questionnaire survey.	West London, England	Six weeks from 1 August 2011	Total n=3157, 60.4% female, 39.6% male	5
44	Spencer, K., *et al.,* Journal of Medical Internet Research, 2016. 18(4): p. e66.	Qualitative: focus groups and interviews.	Salford, England	Not stated	Total n=40, 58% female, 43% male, Ages: 23–88	4
45	Stevenson, F., *et al.,* Family Practice, 2013. 30(2): p. 227–232.	Qualitative: focus groups and interviews	Not stated	Not stated	Total n=57, Patients (n=50), Staff members (n=7).	4

### Quality assessment

Studies’ quality scores ranged from 3 to 6 out of a possible 6, scores of individual studies are shown in Table 1. Two studies which otherwise met inclusion criteria were rejected on the basis of quality and do not appear further in the results ^46, 47^.

### Themes elicited from the studies

The seven themes identified in and elicited from the studies were: Knowledge and Awareness of Electronic Records; Willingness to Share; Privacy; Trust; De-identification and Consent Preferences; Routes to Securing Trust; and Demographic Differences. The contribution of each study to each theme is shown in Table 2.

**Table 2.** Contribution of Studies to Themes.

Study	Knowledge and Awareness of Electronic Records	Willingness to Share Data	Privacy	Trust	De-identification and Consent	Routes to Securing Trust	Demographic Differences
Audrey *et al.* 2016		X	X			X	
Baird *et al.* 2009		X	X	X		X	
Barrett *et al.* 2006	X	X	X				X
Buckley *et al.* 2011	X	X			X		X
Campbell *et al.* 2007					X		
CM Insight and Wellcome Trust 2013	X	X	X	X	X		
Clerkin *et al.* 2013	X	X	X				
Grant *et al.* 2013		X	X	X		X	
Haddow *et al.* 2011		X	X	X		X	
Hays & Daker-White 2015	X	X	X	X	X	X	
Hill *et al.* 2013	X	X	X	X	X	X	
Ipsos Mori, MRC 2007	X	X	X	X	X	X	X
Ipsos Mori, MacMillan, CRUK 2016		X	X		X		X
Ipsos Mori, Wellcome Trust 2016 One Way Mirror	X		X	X		X	X
Ipsos Mori, Wellcome Trust 2016 Monitor Report 3		X					
Luchenski *et al.* 2013		X					X
Papoutsi *et al.* 2015		X	X	X	X		X
Riordan *et al.* 2015	X				X		X
Spencer *et al.* 2016	X		X			X	
Stevenson *et al.* 2013		X	X	X	X		

### Knowledge and awareness of electronic records

Generally, knowledge of the content and electronic collection of GP records among respondents was high. One quantitative study reported that a moderately high proportion of respondents at 59% had prior awareness of EHRs ^43^. Another quantitative study reported that levels of understanding of the information recorded by GPs were high without giving exact numbers ^30^. One qualitative study reported that across groups, participants had a good awareness of the kind of information that usually held in general practice records ^4^. Nevertheless, participant awareness of specific uses of routinely collected patient data was low. For instance, two quantitative studies reported that 82% ^29^ and 80% ^39^ of the general public had not heard of the National Cancer Registry, while another study reported that patients were not only inadequately informed about their right to opt-out of Care.data, but were also unaware of the project ^35^. Two studies indicated that understanding of medical research using patient data was low ^32, 37^, while another suggested that participants were unaware of how their data was currently used ^36^. Another demonstrated limited public grasp of a range of concepts related to patient information use, such as de-identification, data science, the benefits of aggregate data, and the role of private companies in the healthcare system. People with lower understanding of these issues were more likely to have concerns about commercial access to health data ^39^.

### Willingness to share

In many of the studies, participants expressed willingness to share their EHRs for secondary purposes like research, policy and planning, despite the range of concerns discussed below. Among the quantitative studies, support for sharing patient data with researchers was reported at 68.7% ^30^, 77% ^40^, 81.4% ^42^, and 83% ^37^. In qualitative studies, participants identified willingness to share their EHRs for secondary purposes with the “common”, “greater” or “public good” ^4, 27, 28, 32, 37^; “social responsibility” ^44, 45^; “altruistic attitudes” ^28^; and “giving something back” ^33^ to “other people” or “future generations” ^4, 28, 34– 36, 44^. For example, in one study it was stated:

I’m saying yes because I think there is a greater good. (Participant 1, Group 2, ^36^)

Such reasoning was largely predicated on the understanding that medical research using EHRs could lead to benefits such as the improvement of healthcare services, or innovations in the diagnosis and treatment of disease. For example:

I think if you are going to do something, eczema, allergies, something that affects one in five people you need the huge samples in order to do it. (Patient Interview L2, ^45^)

And:

. . . because you never know where research is going to go. You don’t know where some brilliant young scientist’s mind linking up different things, you know. And you cannot put a halt on that, a break on that. (Di, Focus Group 1, ^34^)

Moreover, it was also understood that using EHRs might be a better way of doing and facilitating research:

. . . I mean it’s a better system than it is at present, because you are going to get 100% response that way or near enough and the present system is that the GPs put out things on spec to people that may want to join this thing and they may get a very low return. (Male, Patient Focus Group 3, ^33^)

From these studies, the “common good” appeared to consist of the collective public health benefits brought about by the improvement of the services, practices and methods of healthcare through secondary uses of data. Willingness to share appears connected to idea of an individual having a personal responsibility, obligation or duty to help bring about this common good:

Once you have been in receipt of the excellent kind of care and treatment that I’ve had, I think you have a social responsibility that if you can help the next generation by having your information provided to the researchers to [do] some good. (Focus Group 3, ^44^)

### Privacy

Despite the general willingness to share EHRs for secondary purposes, many qualifying concerns were raised by participants ^27, 28, 32– 39, 42, 44, 45^. This suggests that although the sharing of EHRs is largely seen as being for the overall common good, participants believe that it also has the potential to create new risks, and increase existing ones. The various perceived risks involved in sharing EHRs were well described by participants. These included routes to harm like hacking ^35, 42^, unintentional data leakage or loss ^35^, unauthorised access ^42^, access without explicit consent ^27^, errors in medical records ^42^, re-identification ^34^, aggregating data to a group’s disadvantage ^34^, and access, use and governance of data by the government ^34^. Participants also listed perceived harms as a result of adversaries gaining access to data, these included: identity theft ^42^, unnecessary stigmatising judgements in clinical settings ^42^, consequences for employment, pension eligibility, or insurance costs ^4^, social discomfort and community embarrassment ^4^, and the use of EHRs for financial gain ^36^. The breadth of this list demonstrates the structural complexities of the particular, concrete situations which study participants imagine may arise from the misuse of their data. Several studies connected these risks and the concept of privacy ^4, 27, 28, 32, 39, 42, 44^. Privacy was generally conceptualised by participants as a process of control:

Seemingly radical idea: let PATIENTS control who can access their personal medical data! #caredata. (Twitter user, ^35^)

Participants frequently identified two key elements that could be determined in relation to their information. The first was whether information is revealed to, or accessed by another party:

My concern is exactly that: who has access to my files and how can we make sure that only those I want to have access would have access? (Focus Group 12, ^42^)

A second element concerned how this information should be used, or analysed after it being revealed to another party:

At the end of the day, it’s not who has access to it all, it’s how they use it, I think is the main concern for us, for everybody. . . how they use it. (Person with MS, Focus Group 7, ^28^)

These two factors were necessary components in identifying what was and was not acceptable when it came to unlocking the potential of patient data.

### Trust

Views on storing and using patient data were linked to the kind of trust or distrust the public had in an organisation or individual using or accessing the data.

You have to trust people. (Fiona, Focus Group 2, ^34^)

Where participants distrusted organisations who would handle their data, this generally occurred along two lines:

1. Distrust of a party’s ability, or *competence*, to ensure data security.
2. Distrust of a party’s *motivations*.

In terms of a party’s competence, participants were likely to agree that a particular party could store and use their data in principle, but were concerned that they are not able to guarantee the level of security required by such personal data due to institutional incompetence. One such party was “the NHS” ^36, 42^. For example, in one study a majority of respondents (71.3%) voiced doubts about the ability of the NHS to guarantee the security of EHRs, yet 53.5% of those respondents would nevertheless support the development of a national EHR ^42^. On the incompetence and inefficiency of the NHS, participants stated the following things:

I just have very little faith in the way that the NHS handles databases. I don’t think it’s got a very good record. . . (Focus Group 3, ^42^)
Always thought that [the NHS] would mess it up (Focus Group 11, ^42^)
#NHSPatientdata scheme handling a ‘masterclass in incompetence’ #CareData #NHS [link] [link]. (Twitter user, ^35^)

However, in the some qualitative studies, participants expressed a generalised trust towards the NHS, especially when concerning GPs:

I mean I can trust the doctors and all . . . but other people, no. Once it leaves the NHS, I’d be wondering where it’s going and who’s looking at it. (Participant 19, ^44^)
. . . once it goes out of the NHS, the NHS have no control over it whatsoever. (Di, Focus Group 1, ^34^)

Nevertheless, and perhaps surprisingly, participants tended to say that the data would be safer in the hands of the NHS or a public sector organisation, and that private companies were less likely to be as diligent in their handling of it ^39^.

When it came to an organisation’s motivation, there was a strong sense that any access and use of the data must be for the good of the individual patient or the common good of the public. Many studies indicated that any kind of data handing for private interests would be unacceptable ^32– 36, 42, 45^. In terms of the possible consequences, a recurring theme was that if a party had the wrong competences or motivations, this could lead to substantial harm on both an individual or collective societal level. For instance, as the following quote illustrates, it was identified that the private profit motivations of insurance and marketing companies could lead to harms on an individual level:

One of my fears was if it somehow goes astray from there and somebody, for instance, like insurance companies, get a hold of it they could use it to their advantage and the patient’s disadvantage. (P2, Focus Group, ^45^)

However, direct harm to individuals is not a necessary factor in determining the wrongness of certain motivations. It was also indicated that even if no particular individual is disadvantaged, allowing those with private interests to access public data can constitute a collective harm. This is because there is a strong sense that data should only be used to benefit either individuals:

Financial gain comes into it then so why should you then let them look at your records? They are going to gain out of it and you’re not. . . (Participant 2, Group 2, ^36^)

Or, the public at large:

If there was a large commercial company. . . [that] had free and easy access to people’s medical records I don’t think that would be right. It would further their research into the particular drug or treatment, but it’d also further their profits that would be wrong. But if it was for medical research for everybody then that would be different. (Participant 6, Group 3, ^36^)

Despite this firm belief, several of the studies indicated a tension in the status of pharmaceutical companies whose products are indispensable to medicine and the health of populations, but which ultimately operate in a profit driven capacity ^28, 33, 36, 37, 42^. As Grant *et al.* ^33^ write, this leads some participants to see the involvement of pharmaceutical companies as a “necessary evil”.

This dimension was further discussed in the grey literature which revealed a more nuanced picture regarding public opinion towards the commercial uses of data. Support for commercial access to patient data raised from 54% to 61% when taking into account the possibility of new treatments being discovered ^39^, and participants were indifferent to who conducts research so long as the objective is to increase knowledge around the causes and cures of ill health ^32^. This suggests that participants recognise that not all commercial uses of data are done from purely privately interested motivations, but that at least in part can involve public motivations too. In explaining the apparent reluctance of the public to accept certain private interests so as to ensure public benefits, one study identified that participants did not currently feel that they could evaluate the motivations of commercial organisations who would use the data, which created an unclear conception of what the public could stand to gain through these uses of data. As a result, participants tended to fall back into wider assumptions, personal beliefs and prejudices regarding private companies ^39^.

### De-identification and consent preferences

In the quantitative studies, 67.5% ^30^ of respondents in 2011 and 91% ^43^ of respondents in 2015 were clear that although it was fine for researchers to access their EHRs, they still expected to be asked for consent when their identifiable data was accessed for secondary purposes. However, there was less consensus over de-identified data, with 83.7% ^30^, 51% ^31^, and 49.3% ^43^ of respondents reporting willingness to share or agreement that de-identified patient data could be extracted without consent. Reasons for concern around de-identification also emerged in the qualitative studies where participants questioned what would qualify as identifying information ^42^, whether de-identification could be achieved effectively ^37, 42^, whether it was sufficient for the elimination of consent ^27, 36^ and highlighted the risks of re-identifying individuals ^32, 35^.

Several studies also indicated substantial concerns about the opt-out rather than opt-in model of consent which was proposed in schemes such as Care.data ^35, 45^, while others noted that participants generally thought about consent along opt-in lines when asked for their opinions ^27^. Participants expressed worries about whether people would really understand the concept of opting-out ^45^. They also criticised opt-out on the basis that it was unethical and illegal ^35^. However, in one quantitative study 52% of the general public supported the opt-out method of collection for the National Cancer Registry ^38^, while a minority of participants in another study acknowledged that opt-out might be a better option given the impracticalities of opting-in ^37^.

The problem of selection bias and its connection with consent arrangements was explored in three studies ^27, 36, 42^. In two studies, some participants identified the potential for bias if the information which was gained was neither accurate nor balanced ^27, 42^:

If they’ve got mental health illness then. . . that might affect their willingness, so it might be hard to. . . gather enough information. I think that might be biased. . . (Male ID47, ^27^)

Participants also recognised that larger, more representative samples could be gained by an opt-out process:

You are going to get 100% response that way, or near enough and the present system is that the GPs put out things on spec to people that may want to join this thing and they may get a very low return. (Male, Patient focus group 3, ^33^)

This prompted discussion in one study about the importance of mitigating the requirement of consent by de-identifying information:

There is certain situations where you might be able to, it might be acceptable to ask or it might be acceptable just to go ahead and get it—as long as it wasn’t directly linked back to you as a person, it would be alright. . . (Female, ID6, ^27^)

In another study ^36^, after receiving presentation about selection bias, participants recognised the difficulties faced by researchers. Interestingly, when asked if this information had changed their opinion about using health data without consent, several participants out of the group who at first indicated reluctance, reported that they had indeed changed their minds. A quantitative study showed that a substantial minority of respondents (20%) ^37^ believe that consent may not be needed if it is not practical to obtain.

### Routes to Securing Trust

Across studies, participants identified several different infrastructure arrangements which could increase willingness to share patient data for secondary purposes and trust in their use for public benefit. Participants indicated that no single organisation should be responsible for deciding who could access and use their EHRs, rather a committee of stakeholders was called for, including Caldicott Guardians, research consultants, members of the public, GPs, social services staff, charities, funders, and patients. ^28, 34^. It was also felt that greater transparency was needed in regards to safeguarding processes and data sharing arrangements ^35, 44^, including stiff penalties or fines for misuses of data ^35, 39^; the publication of results ^39^; clear guidelines and laws to regulate access and use of data ^35^; and, regulators and parties accessing data to be held to high standards ^39^. Several studies also indicated that participants wanted a better understanding about the nature of EHR initiatives, medical research ^37^, the purposes and benefits of using data ^33, 37^, de-identification and aggregation ^39^, and also why in some situations consent might not be practical ^39^. More generally, participants wanted the security of records to be ensured ^33, 39^; for private profit to be capped ^39^; and denial of third party access ^39^. In several studies, participants also indicated their preference to retain granular control over the data in their EHR using an explicit opt-in consent scheme, the right to withdraw at any time and ability to tailor sharing preferences ^28, 33, 35, 44^.

Despite the breadth and diversity of participant suggestions to increase trust, it might be that no single, or any specific combination of strategies will amount to a gold standard of acceptability or social licence. One study found that no particular safeguard made sharing data with commercial companies any more acceptable than any other ^39^. However, in the same study, participants were significantly less likely to endorse sharing data without any safeguards (49% agreed) compared to with safeguards (56–64% agreed, depending on the safeguard). This suggests that the precise nature of the safeguard may be less important to improving willingness to share than knowing that there are safeguards in place.

### Demographic differences

We aimed to ascertain whether the included studies indicated a level of heightened concern, worry or fear among one or more specific social groups and we restricted this analysis to quantitative studies which could enable such contrasts. Although participants were asked a variety of different questions across each survey, we evaluated responses on the basis of whether they indicated an overall negative or positive attitude towards the sharing of EHRs for secondary purposes such as research. For example, in Papoutsi *et al.* ^42^, participants were asked if they would be more worried about the security of their information if it were part of a national EHR register, while Buckley *et al.* ^30^ asked if they would allow their EHRs to be provided to researchers without their explicit consent. Despite the differing approaches of these questions, we concluded that a response indicating more worry about security, and one indicating less likelihood of granting researchers access without explicit consent, were comparative insofar as they represented a negative attitude towards sharing of EHRs.

Within quantitative studies, findings were reported across a whole range of demographic differences. Between studies, comparison could only be made between age range, levels of education, and ethnicity. We found conflicting findings in all three of these categories. We found evidence that both younger people and older people would favour sharing their data, that people with lower levels of education were both more and less likely to agree to sharing without consent, and that people of non-white ethnicity were both more and less likely to support EHRs and think of them as secure. For a full break down of the demographic results, see Table 3.

**Table 3.** Study findings on Demographic Differences.

Group	Indicative of Negative Attitude	Indicative of Positive Attitude
Age	Compared to those aged 25–34, respondents between the ages of 35–64 were more likely to report they would be worried about the security of their records as part of a national EHR ^42^.	Increase in age by each 10 year increment was significantly associated with an increased likelihood of reporting that any info can be provided to researchers without asking for consent ^30^.
	Compared to those aged 25–34, respondents over 35 years old were more likely to report less confidence in the ability of NHS security and were less likely to report that EHRs were equally or more secure than paper records ^42^.	Older people (55–64, 65+) were more likely to find a drug company conducting research into the unwanted side effects of a drug using deidentified data to be more acceptable than younger people (16–24, 25–34, 35–44, 45–54) ^39^.
	Older people were increasingly more likely to report that they would not be in favour of a national EHR compared with 25–34 year olds ^41^.	Those aged 55–64 tended to agree that research should be conducted by commercial organisations if there is a possibility of new treatments being discovered in comparison to 16–24s and 35–44s ^39^.
		In the general public, support for the opt-out collection method was higher in over 55s (58%) than 18–34 (49%) and 35–54s (49%) ^29^. Those over 55 were more likely to say to say that they would allow their data to be used for medical research compared to those aged 16–24 ^37^.
Education	Respondents with lower educational qualifications were more likely to expect to be asked for explicit consent before their deidentified records were accessed ^43^.	Compared with participants with higher degrees, individuals with no academic qualifications were less likely to say that they would worry about security if their record was part of a national EHR ^42^.
		Compared with completion of third level education, completion of only primary level education was associated with increased likelihood of reporting that any info can be provided to researchers without asking for consent ^30^.
Socioeconomic Status	Those of a lower socioeconomic status were more likely to be concerned about privacy ^29^.	Those in the lower socioeconomic group DE (43%) were more likely to support companies using health data collected in the NHS to help target health products at different groups of people ^39^.
	Those in socioeconomic groups C2 and DE were less likely than those in AB and C1 to view the use of health data as having a potential benefit to society ^32^.	
	Those in the lower socioeconomic group DE were less likely to say they trusted a variety of people with their health data; say that the advantages outweigh the disadvantages of using health data in research; and say that researcher can use data without prior consent than Abs ^37^.	
	Those in socioeconomic groups C1 and C2 were less likely than ABs to allow their health data to be used ^37^.	
	Those in socioeconomic groups DE (46%) were less likely to support commercial organisations to undertaking health research with health data than AB (62%) ^37^.	Those in socioeconomic groups DE (26%) were less likely to support commercial organisations to undertaking health research with health data than AB (30%) ^37^.
Ethnicity	Black British respondents were more likely to say they would not support the development of a national EHR system compared with White British respondents ^41^.	Compared with White British groups White non-British, Asian, British Asian, Black-African, Caribbean, and British Black groups were more likely to say that EHRs are as secure, or more secure that paper records ^42^.
	Respondents identifying as belonging to an ethnic group other than White British were more likely to expect to be asked for explicit consent before their deidentified records were accessed ^43^.	
	Those whose ethnicity was not White British were more likely to be concerned about the invasion of privacy ^29^.	

## Discussion

We found that knowledge of the content and collection of patient data in EHRs was reasonably high, but knowledge about the secondary uses, such as data sharing for research, was low. Nevertheless, when asked, participants were generally willing to share their data for the “common good”, subject to safeguards. Willingness was qualified with concerns about privacy which participants generally equated with the idea of control. This conceptualisation of privacy as control closely corresponds to the idea that informational privacy is the ability of an individual to determine for themselves what happens with certain information relating to them ^48, 49^. This particular definition has attracted criticism insofar as it difficult to capture what constitutes “certain” information ^50^. Within the legal and philosophical literature it is generally accepted that what constitutes an individual’s determination is whether or not information is communicated to other parties, however, our analysis suggests that the public also believes that their privacy can be violated not just in the sharing of their information, but in the subsequent use of that information too (e.g. using personal information for profit). 

Participants feared adverse outcomes less when they trusted both the motivation of research organisations to conduct research for the common good, and the competence of organisations to handle the data safely and without compromise. When evaluating opinions on consent mechanisms, findings suggested that educational and deliberative research into public opinion may provide different answers from snapshot surveys. This is because after weighing up a range of issues involved, participants could often see the benefit to research quality of opt-out schemes. Results suggested a range of mechanisms to increase public trust, and the overarching theme here was transparency of motivation, data handling and data flow.

### Core Ethical Principles

The foundational moral principles which Beauchamp and Childress ^14^ identify as paramount to governing biomedical practice, and which Belmont identified as important for medical research ^15^ can be used as a lens for understanding and interpreting these findings. Where we find that public reasoning maps to these basic principles, it can be inferred that these core ethical principles are a constituent part of non-specialist thinking about the ethical practicalities of healthcare and medicine. This in turn identifies these ethical principles as a suitable structure for guiding reasoning on future data sharing challenges. 

For instance, the included studies indicate that there is a widespread willingness to share EHRs for secondary purposes, in principle. This willingness was held on the basis that, using and accessing data in such a way can bring about benefits which are in the interests of all individuals, or in other words, the “common good”. The basis of this belief may be the general expectation that if members of the public can contribute to the welfare of each other by sharing data, then they feel a moral obligation to do so. We could reinterpret this as the principle of beneficence, which urges us to act, where we can, to promote good.

Willingness to share data rarely led to unqualified support of the schemes designed to enable secondary use. Support was withheld because, in practice, it was felt that key values would not, or could not, be ensured, thus bringing with it the risk of individual and collective harm. The public might feel justified in objecting to irresponsible, or insecure use of data because it is likely to cause individual harm; a direct violation of the principle of non-maleficence. Similarly, the use of data for private gain may be said to be in violation of the principle of justice because it is generally unfair to exploit something for reasons other than what it was intended for. Finally, the use of patient data without transparency or consent may be seen to violate the principle of respect for autonomy.

### Strengths and limitations

We conducted a wide search and sifted a huge number of papers, including grey literature reports. The search was challenging due to wide range of terms used within the literature for secondary data usage, and for expressing the concept of public opinion and attitudes. We cast a wide net and spent time excluding papers, and believe this review encompasses all available research meeting our criteria up until the search was conducted. Our findings were deliberately limited to UK and the Republic of Ireland to create a manageable, relevant and comparable body of literature. This enabled us to look for underlying principles for publics exposed to a particular type of healthcare system, but findings are obviously only applicable within these contexts. There may also have been differences between UK and Irish respondents due to differences in these healthcare systems. Both systems have a general practice plus hospital system. However, in the UK, all GP and hospital visits are free at the point of use, whereas in Ireland, around two thirds of the population must pay a fee for GP or hospital care. This financial transaction may influence how patients perceive ownership and use of their medical data, although we found no literature on this.

Synthesis of results was also challenging as there was a wide range of study types, using different methods. The small, convenience samples and low response rates in the majority of studies is also likely to have introduced bias into the findings, as it is probable that only members of the public most interested in the issues consented to take part in the research. This means that each study likely represents a narrow range of views, and views expressed may have been influenced by the means of data collection. It is not clear how this might have affected results across the whole range of studies, but it is likely that the themes and views represented here are not a complete picture of the public’s opinions. This may have contributed to the inability to find systematic differences in views between demographic groups. Additionally, certain research questions of particular interest were not asked of participants and therefore our understanding of public opinion is still limited. One example of this is whether the use of medical text (in contrast to structured data in medical records) elicits specific privacy concerns for the public.

Our analysis was informed and influenced by our respective backgrounds in philosophy, psychology and epidemiology. While attempting to be data-led, we must acknowledge that we may not have been wholly neutral in approach. However, our review highlights similar themes to Aitken *et al*. ^13^, suggesting a consistency with other syntheses in this area.

### Future directions

This review demonstrates and makes explicit the extent to which public attitudes to sharing health data are based on reasoning in line with established bioethics principles. Decision makers, who evaluate data-sharing proposals can therefore draw on an explicit framework of ethical principles to address challenges around the sharing of patient data. It is becoming increasingly accepted that the use of patient data for research or for the development of novel healthcare technologies should be supported by a social licence to operate ^9^. According to social licence theory, the public expect that organisations who are instituting potentially controversial schemes (such as patient data sharing) will go beyond the requirements of formal regulation and adhere to voluntary codes of trustworthy behaviour ^51^. Where the public are satisfied that the motivations of the organisation are trustworthy, they confer a “social licence” to operate. It has been hypothesised that previous patient data-sharing initiatives, such as Care.data, have failed to secure public support because they lacked a social licence for their operation ^9^. 

Public views are complex, and interpreting them to guide policy can be difficult. A simple and explicit framework may act as a focussing lens, reducing complexity by pulling out underpinning moral principles in participants’ views. Establishing core values held by the public may facilitate identification of the types of safeguards which could help to secure a social licence for sharing patient data. We make recommendations about how public views could fit into the four tenets of the Beauchamp and Childress framework, and could be used to guide decision makers or regulators. We phrase these suggestions as guiding questions, which could be asked of research proposals by ethics committees and regulators.

1. Do the methods of data collection and usage in the proposal **respect individual patient autonomy**? (Respect for Autonomy)

Patient autonomy can only be achieved if inclusion of stakeholders and transparency of motivation and data flows are assured at all parts of the research process, from study design, through ethics approvals, to analysis and interpretation of results ^52^. It is also essential that individuals have the possibility to opt-out of any data collecting schemes. Notably, the opt-out is only a meaningful way of ensuring individual autonomy if transparency of data usage, and stakeholder inclusion, is guaranteed. This combination (opt-out plus full transparency) is also the public’s preferred approach ^53, 54^, and is thus vital for maximising public trust, and securing a social licence, for any initiative. One example of operationalising a transparent patient opt out was launched by the NHS in the UK in May 2018. Known as the National Data Opt-Out ^55^, it was originally recommended and designed by the UK National Data Guardian’s Office.

2. Are the objectives and the intended outputs primarily concerned with contributing to the **public good**? Do they have clear scientific value? (Beneficence)
3. Is any agreement between the NHS and organisations providing analytics (private or public) **fair and just**? (Justice)

One almost universal finding was that the public generally support research using patient data if the research is for the common or public good. They tend not to support research using patient data which enables private companies to increase profits. Thus, to retain a social licence, ethical bodies and regulators must evaluate proposals on the basis of their intended aims and whether they contribute significantly towards the common good. The engagement of industry and private companies to provide data analytics will be crucial to maximise benefits from patient data in the future. Where private companies are involved, there should be clear and transparent communication to all stakeholders about how a fair settlement has been negotiated, so that the public and patients benefit from the data usage as well as the company. Benefits which come from patient data research should additionally be publicised and communicated, so that these common gains become part of the public consciousness. Examples of good practice in this sphere can be seen in the UK Farr Institute ^56^ and the Wellcome Trust Initiative “Understanding Patient Data” ^57^.

4. Could granting access to the data, or granting a particular use of the data, lead to **individual or collective harm**? (Non-maleficence)

Participants in the studies we reviewed articulated a range of harms that they fear could arise from re-use of patient data. Possible risks may include individual harms, such as re-identification and discrimination from insurance companies or government agencies. However, ethical bodies and regulators should also consider the risk of collective harms from pursuing certain research agendas. For example, failure to achieve fairness or transparency in data-sharing agreements may result in a loss of public trust in the endeavours of research, or in public institutions’ policies on keeping data safe. Such a loss of public trust would put at risk any gains made in securing a social licence for sharing patient data. In addition, infrastructure put in place to safeguard patient privacy must be made transparent to stakeholders to increase trustworthiness. These may include high standards for data storage security, restrictions on data linkage where necessary, evaluation of analytical methods, and consistently applied sanctions for any breaches in data security.

## Conclusions

Our interpretation of a range of studies of public views suggests that the public generally support the use of patient data for research purposes. However, the public demand that projects of this nature are conducted in a secure way to prioritise privacy, and minimise individual and collective harm; that projects set research objectives (or negotiate agreements with third parties) which are primarily concerned with contributing to the common good; and that they do this in a spirit of transparency and inclusivity of stakeholder views. So long as these values are maintained, it is likely that the majority of the public will willingly share their patient data for research purposes.

We have shown that public thinking about the privacy issues around sharing patient data for research maps onto established biomedical ethical principles, and such understanding may help researchers or regulators to identify how the public comes to confer a social licence on patient data research. These core principles can be developed to frame guidance for data custodians, regulators and researchers when planning or approving research projects using patient data.

## Supplementary material

Supplementary File 1: PRISMA checklist.

Supplementary File 2: PRISMA flowchart, showing the number of records identified, included and excluded.
