# Supplementary material for: “Giving something back”: A systematic review and ethical enquiry into public views on the use of patient data for research in the United Kingdom and the Republic of Ireland
Source: Wellcome Open Res. 2019 Jan 17;3:6. Originally published 2018 Jan 16. [Version 2] doi: 10.12688/wellcomeopenres.13531.2 (PMC6402072; doi:10.12688/wellcomeopenres.13531.2)
Supplement: Supplementary file 2 [file wellcomeopenres-3-16368-s0001.tgz › f02465a4-fb92-484a-a545-3706cb4ce7a9_Supplementary_file_2_new.pdf]

PRISMA 2009 Flow Diagram

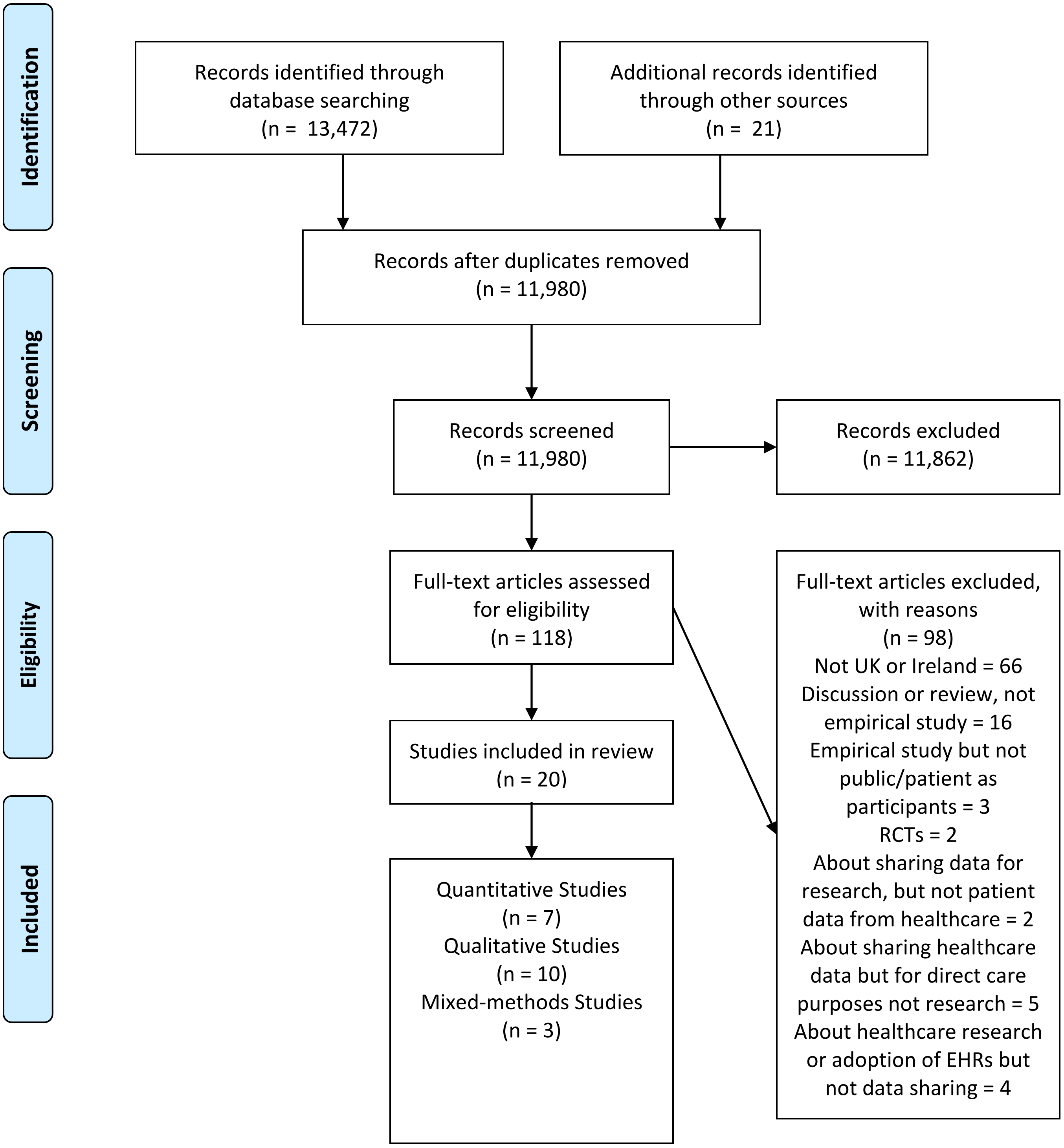

From: Moher D, Liberati A, Tetzlaff J, Altman DG, The PRISMA Group (2009). *P*referred *R*eporting *I*tems for *S*ystematic Reviews and *M*eta-*A*nalyses: The PRISMA Statement. PLoS Med 6(7): e1000097. doi:10.1371/journal.pmed1000097

For more information, visit www.prisma-statement.org.
