# Supplementary material for: “Giving something back”: A systematic review and ethical enquiry into public views on the use of patient data for research in the United Kingdom and the Republic of Ireland
Source: Wellcome Open Res. 2019 Jan 17;3:6. Originally published 2018 Jan 16. [Version 2] doi: 10.12688/wellcomeopenres.13531.2 (PMC6402072; doi:10.12688/wellcomeopenres.13531.2)
Supplement: Peer review history

# Peer review: Reviewer response for version 2

**DOI:** 10.21956/wellcomeopenres.16368.r34595

**Published:** 2019-03-04

**Authors:** Chrysanthi Papoutsi

**Affiliations:** 1 Department of Primary Care Health Sciences, University of Oxford, Oxford, UK

No further comments - the authors have done an excellent job in addressing issues raised previously.

I have read this submission. I believe that I have an appropriate level of expertise to confirm that it is of an acceptable scientific standard.

# Peer review: Reviewer response for version 2

**DOI:** 10.21956/wellcomeopenres.16368.r34596

**Published:** 2019-02-18

**Authors:** Sarah Cunningham-Burley

**Affiliations:** 1 Usher Institute, Edinburgh Medical School, University of Edinburgh, Edinburgh, UK

The authors have made careful revisions.  The paper is clearer and more cogently argued. The review will be useful to all those engaged in research with patient medical data and its governance:  the challenge now is the extension to health-relevant data and to understand and benefit from diverse approaches within the UK devolved administrations, particularly Scotland with its Data Linkage Strategy.

I have read this submission. I believe that I have an appropriate level of expertise to confirm that it is of an acceptable scientific standard.

# Peer review: Reviewer response for version 1

**DOI:** 10.21956/wellcomeopenres.14693.r29910

**Published:** 2018-02-12

**Authors:** Chrysanthi Papoutsi

**Affiliations:** 1 Department of Primary Care Health Sciences, University of Oxford, Oxford, UK

This paper on public opinions about the use of patient data provides a comprehensive overview of relevant studies. The synthesis covers significant ground in drawing together findings from a mix of study designs. Despite long-standing debates in this area, the topic continues to be of importance for policy and practice. Please see some suggestions for improvement below.

 Perhaps revise some of the full quotes provided in the introduction so that the text flows better.

 Can you please elaborate on how this review differs from existing systematic reviews on this topic and its contribution to knowledge?

 The paper follows systematic processes for literature searching and screening. The approach to analysing the data has been methodical. More explanation is needed on whether the paper has followed any established approaches for systematically reviewing mixed methods, secondary data (e.g. see Dixon-Woods et al. (2005) ^1^ or Thomas and Harden (2008) ^2^) and if not, why not. Could you please provide more details on the following: ‘we undertook a deeper analysis of meaning within findings guided by both metasynthesis principles ^20^ and established principles of bioethics ^9^’.

 Please elaborate on the application of the Mixed Methods Appraisal Tool (MMAT) for assessing quality across different study designs. The findings section draws heavily on qualitative data, however, these studies tend to be ranked lower in terms of ‘quality’ – are studies being prioritised based on a hierarchy of evidence or are they judged based on the merit of each study design, and what does this mean for the topic studied here? How has the study using Twitter data been assessed for quality and inclusion (e.g. is it clear whether participants are from the UK or Ireland for example)?

 Please revise the PRISMA diagram to clarify which studies were only quantitative, which were only qualitative and which were mixed methods (these numbers are provided correctly in the text).

 It is difficult to interpret syntheses of results presented as a list of percentages e.g. ‘Among the quantitative studies, public support for a national EHR system was reported at 62.5% ^36^, 62.47% ^35^, and 81% ^23^, while support for sharing information in general was reported 73% ^32^.’ What do the authors mean by national EHR system, what does ‘public support mean’, what does ‘sharing information’ mean and what do these percentages refer to? Of course these terms are artifacts of the studies reviewed here but it would be helpful to provide more context for the reader who has not seen the original studies, when presenting percentages across the document.

 There are differences in the healthcare and EHR systems across the UK and Ireland – it would be worth reflecting on this when synthesising results from different studies.

 Academic literature on privacy may help clarify some of the nuances around control and self-determination (e.g. when it is mentioned that ‘Privacy was widely conceptualised as a process whereby an individual determines for themselves what happens with the information relating to them.’) Further use of background literature and theory could inform the analysis.

 It would be useful to elaborate on the use of the Beauchamp and Childress framework. Are there any pre-existing applications of this framework to study patient attitudes? How do the nine questions used for data analysis fit with the Beauchamp and Childress framework? Was the framework used as part of the analysis or as a lens to discuss the findings? If the latter, more extensive and critical discussion is needed – perhaps reflecting on how normative ethical frameworks can encompass the messiness of everyday reality and practice.

 The paper mentions contradictions between studies based on demographic characteristics. It would be useful to reflect on why these differences may have occurred and how qualitative data could help explain them.

 The future directions section needs further development – this links back to the use of the Beauchamp and Childress framework. The authors present 4 questions for policy and practice but may need to further clarify how these would be used, how some of the terms need to be understood (e.g. what constitutes patient autonomy is in itself a challenging topic of philosophical contention) and whether the answer to these 4 questions could ever be straightforward in practice.

I have read this submission. I believe that I have an appropriate level of expertise to confirm that it is of an acceptable scientific standard, however I have significant reservations, as outlined above.

## References

[1] Dixon-WoodsMAgarwalSJonesDYoungBSuttonA: Synthesising qualitative and quantitative evidence: A review of possible methods. *Journal of Health Services Research & Policy*.2005;10(1) : 10.1177/135581960501000110 45-53 10.1177/135581960501000110 15667704

[2] ThomasJHardenA: Methods for the thematic synthesis of qualitative research in systematic reviews. *BMC Med Res Methodol*.2008;8: 10.1186/1471-2288-8-45 45 10.1186/1471-2288-8-45 18616818PMC2478656

# Response

**Published:** 2019-01-04

**Authors:** Elizabeth Ford

**Affiliations:** Brighton and Sussex Medical School, UK

We would like to thank you for these very helpful comments, which have enabled us to substantially improve the paper. We spent a lot of time discussing these insightful comments in order to best make improvements to the manuscript. We detail point by point below how we have addressed each comment. We have highlighted our changes in our revised manuscript in red font.

**1) Perhaps revise some of the full quotes provided in the introduction so that the text flows better.**

We have completely updated the paragraph with the quotes, to reflect new changes in the law due to GDPR. No quotes are included in the new paragraph (page 3). 

**2)** **Can you please elaborate on how this review differs from existing systematic reviews on this topic and its contribution to knowledge?**

We have added a new section on this on page 4 in the “Striking a balance” paragraph. We have described how by using a well-recognized ethical framework we can draw underlying themes from the results which may help to organize policy.

**3) More explanation is needed on whether the paper has followed any established approaches for systematically reviewing mixed methods, secondary data (e.g. see Dixon-Woods et al. (2005) or Thomas and Harden (2008)) and if not, why not. Could you please provide more details on the following: ‘we undertook a deeper analysis of meaning within findings guided by both metasynthesis principles and established principles of bioethics’.**

We have included a new section on data synthesis in the methods section page 6, outlining how we used a thematic analysis to interpret the data (a method recommended by both Dixon-Woods et al. and Thomas and Harden). We have extended this section to explain how the Beauchamp and Childress framework informed our analysis.

**4) Please elaborate on the application of the Mixed Methods Appraisal Tool (MMAT) for assessing quality across different study designs. The findings section draws heavily on qualitative data, however, these studies tend to be ranked lower in terms of ‘quality’ – are studies being prioritised based on a hierarchy of evidence or are they judged based on the merit of each study design, and what does this mean for the topic studied here? How has the study using Twitter data been assessed for quality and inclusion (e.g. is it clear whether participants are from the UK or Ireland for example)?**

We treated insights from qualitative and quantitative studies as having different roles but equal value in our enquiry, and therefore if they met MMAT criteria we did not further differentiate between methodologies in terms of a hierarchy. We have explained this on page 6.

**5) Please revise the PRISMA diagram to clarify which studies were only quantitative, which were only qualitative and which were mixed methods (these numbers are provided correctly in the text).**

 We have revised the PRISMA diagram as requested (supplementary file 2)

**6) It is difficult to interpret syntheses of results presented as a list of percentages e.g. ‘Among the quantitative studies, public support for a national EHR system was reported at 62.5% ^36^, 62.47% ^35^, and 81% ^23^, while support for sharing information in general was reported 73% ^32^.’**

We revised the reporting of results in this section, because, when we considered its value within the paper, we found the sentence that the reviewer referred to did not answer any of the outlined research objectives. We now only present evidence on participants’ willingness to share their patient data for research in this section.

**7) There are differences in the healthcare and EHR systems across the UK and Ireland – it would be worth reflecting on this when synthesising results from different studies.**

We have added a paragraph reflecting on the differences in the two systems and how this could influence results. Page 24.

**8) Academic literature on privacy may help clarify some of the nuances around control and self-determination (e.g. when it is mentioned that ‘Privacy was widely conceptualised as a process whereby an individual determines for themselves what happens with the information relating to them.’) Further use of background literature and theory could inform the analysis.**

We have added a paragraph in the discussion on pages 22-23, to describe further literature on the nuances around the conceptualisation of privacy.

**9) It would be useful to elaborate on the use of the Beauchamp and Childress framework. Are there any pre-existing applications of this framework to study patient attitudes?**

We reference the Beauchamp and Childress framework, but note its similarity to other ethical principles such as Belmont. While we have not found such basic principles applied to study patient attitudes (now explained on page 6), we have found them applied to information technology research (Menlo report, now described and referenced, page 4). We used this simple framework to cast a lens on the findings of our review, as a way of sorting and filtering through the complexity of public opinions. A stable and concise framework might enable policy makers and regulators to more efficiently apply stakeholder views to their decision making, thus facilitating securing a social license for research.

**10) How do the nine questions used for data analysis fit with the Beauchamp and Childress framework?**

The nine questions for the data analysis were driven by the problem of data sharing for health research as it manifested itself, rather than our interpretation of the problem through the Beauchamp and Childress framework. They represent the first iteration of our search for themes within the data. We have made this clearer on page 6. Our assimilation of the results was data-driven, and Beauchamp and Childress only used to add the highest levels of interpretation.

**11) Was the framework used as part of the analysis or as a lens to discuss the findings? If the latter, more extensive and critical discussion is needed – perhaps reflecting on how normative ethical frameworks can encompass the messiness of everyday reality and practice.**

We have provided much more clarity on our use of this framework, on page 4. We say: “We use these widely accepted principles as a tool to identify patient reasoning in the analysis, and additionally as a lens to discuss the findings in terms of the newly adopted social license theory for patient data research. Identifying a framework which describes the core moral or ethical values underlying public views may help us to understand approaches to sharing patient data for research that the public will deem as acceptable, and help us to predict the reaction of the public to new data sharing challenges in the future.”

**12)** **The paper mentions contradictions between studies based on demographic characteristics. It would be useful to reflect on why these differences may have occurred and how qualitative data could help explain them.**

In our investigation of differences in views by demographic characteristics, we did not find any replicable trends across quantitative studies. This may be because quantitative studies were limited in their ability to rigorously identify differences, or because such difference do not exist. Therefore we cannot speculate on reasons for differences, because we have not got any firm evidence that these differences exist. We have added a sentence on this to the discussion. Page 24.

**13)** **The future directions section needs further development – this links back to the use of the Beauchamp and Childress framework. The authors present 4 questions for policy and practice but may need to further clarify how these would be used, how some of the terms need to be understood (e.g. what constitutes patient autonomy is in itself a challenging topic of philosophical contention) and whether the answer to these 4 questions could ever be straightforward in practice.**

Many thanks for these suggestions. We have substantially re-written this section.

# Peer review: Reviewer response for version 1

**DOI:** 10.21956/wellcomeopenres.14693.r29907

**Published:** 2018-01-30

**Authors:** Sarah Cunningham-Burley

**Affiliations:** 1 Usher Institute, Edinburgh Medical School, University of Edinburgh, Edinburgh, UK

On the whole this is a clearly presented systematic review (a copy edit is required as there are a few typos) and it reinforces the findings of a similar systematic review that I am co-author on, as the authors note in their conclusion. However, this review included quantitative studies and focused only on UK and Ireland, so the articles included do not fully overlap - the reviews were different in scope. So this is an additional contribution to the literature on public attitudes to data linkage and sharing for health research. 

 The process of the systematic review is delineated well and there is sufficient information on each included article for the reader to be able to access these and also to relate the findings of the review to those articles.  The authors also cite some other relevant literature not included in the review. The authors are appropriately cautious in their interpretation of the findings from various studies, as these are often small scale, limited response rates etc. 

 I have a few concerns about the paper. The authors do not seem to be aware of existing governance structures, carefully developed alongside research on public attidues and legal and ethical analyses. Health is a devolved matter in the UK. They need to read and make reference to the Scottish Government’s Data Linkage Framework ( http://www.gov.scot/Topics/Statistics/datalinkageframework), the Guiding Principles for Data Linkage, and the terms of reference for the Public Benefits and Privacy Panel for Health and Social Care. Perhaps also look at the FARR Institute website to see how this major initiative is promoting safe use of health data for research purposes. There are some key reports that have not been identified by their search that are highly relevant: Public Acceptability of Cross-Sectoral Data Linkage ( http://www.gov.scot/Publications/2012/08/9455); Public acceptability of data sharing between public, private and third sectors for research purposes ( http://www.gov.scot/resource/0043/00435458.pdf); Aitken et al (2011) ^1^.

 These would all help the authors craft more apposite recommendations. 

 A few other points – while supportive of an approach that identifies core principles, I’m not sure that Beauchamp and Childress’ four principles for biomedical research translate as easily as they suggest. I think some reference to emergent frameworks that speak to a social licence might be more compelling and the core principles that might underpin such a license. Public health ethics might help here. 

 A more minor point – the authors start by referring the medical data but really they are focussed on health data – a broader term. I also wonder why they use the term public opinion instead of attitudes. It may be that these terms are used differently in quantitative and qualitative research perhaps, but some justification would be helpful. During the presentation of the findings of the review, they also refer to GP records, the Electronic Health Record, Cancer Registries – maybe clarify what type of records the studies they are referring to – or use the overarching term of EHR. The authors touch on the differences in the way in which public’s views are accessed and I think that point bears further elaboration.

I have read this submission. I believe that I have an appropriate level of expertise to confirm that it is of an acceptable scientific standard, however I have significant reservations, as outlined above.

## References

[1] AitkenMCunningham-BurleySPagliariC: Moving from trust to trustworthiness: Experiences of public engagement in the Scottish Health Informatics Programme. *Sci Public Policy*.2016;43(5) : 10.1093/scipol/scv075 713-723 10.1093/scipol/scv075 28066123PMC5210028

# Response

**Published:** 2019-01-04

**Authors:** Elizabeth Ford

**Affiliations:** Brighton and Sussex Medical School, UK

Many thanks for your helpful comments which have enabled us to substantially improve the paper. We detail point by point below how we have addressed each comment. We have highlighted our changes in the manuscript in red font.

**1) Copy edit is required.**

Thank you, we have thoroughly proofread the paper.

**2) The authors do not seem to be aware of existing governance structures, carefully developed alongside research on public attidues and legal and ethical analyses. Health is a devolved matter in the UK. They need to read and make reference to the Scottish Government’s Data Linkage Framework (http://www.gov.scot/Topics/Statistics/datalinkageframework), the Guiding Principles for Data Linkage, and the terms of reference for the Public Benefits and Privacy Panel for Health and Social Care.**

Thank you for pointing out the regional differences and the link to the Scottish framework. We have replaced this section with a general overview of the new EU GDPR legislation (page 3) and its general implications for the sharing of patient data, with no references made to specific countries’ frameworks or data access policies.

**3) Perhaps also look at the FARR Institute website to see how this major initiative is promoting safe use of health data for research purposes**

We have included reference to Farr, and the Wellcome trust initiative Understanding Patient Data as key exemplars of disseminators of public benefits of patient data research, in the discussion page 25

**4) There are some key reports that have not been identified by their search that are highly relevant: Public Acceptability of Cross-Sectoral Data Linkage (http://www.gov.scot/Publications/2012/08/9455); Public acceptability of data sharing between public, private and third sectors for research purposes (http://www.gov.scot/resource/0043/00435458.pdf); Aitken et al (2011) ^1^. recommendations.**

Many thanks for suggesting these reports. We scrutinised these reports in detail and found they did not meet our eligibility criterion that studies must be about sharing health data in particular and not personal data in general.

**5) A few other points – while supportive of an approach that identifies core principles, I’m not sure that Beauchamp and Childress’ four principles for biomedical research translate as easily as they suggest. I think some reference to emergent frameworks that speak to a social licence might be more compelling and the core principles that might underpin such a license. Public health ethics might help here.**

Many thanks for these suggestions which mirror recommendations from reviewer 1 and have helped us to strengthen the main messages of the paper. We have substantially rewritten the future directions section and the majority of the discussion. We have given more background information on the use of key ethical principles such as Beauchamp and Childress in the introduction, and have related these principles to social license theory throughout the paper.

**6) A more minor point – the authors start by referring the medical data but really they are focussed on health data – a broader term. I also wonder why they use the term public opinion instead of attitudes. It may be that these terms are used differently in quantitative and qualitative research perhaps, but some justification would be helpful.**

Papers used a variety of different terms denoting that they were capturing the thoughts of patients and the public, including: views, perspectives, attitudes, perceptions, opinions, acceptance, awareness, thoughts. We have decided to use generically the term public “views” because this feels like the most general term, and we have made this consistent throughout.

We agree with the reviewers on the need for clarification of the terms medical and health data. We have described the type of data we are focusing on in the methods (page 5), as “electronic hospital records, electronic general practice records, and data extracted from these records, for example cancer registries and national disease databases” and have used the terms patient data or EHRs to represent these data throughout the manuscript. We preferred the term “patient data” to keep our language consistent with public facing initiatives such as the Wellcome Trust “Understanding Patient Data” initiative.

**7) During the presentation of the findings of the review, they also refer to GP records, the Electronic Health Record, Cancer Registries – maybe clarify what type of records the studies they are referring to – or use the overarching term of EHR. The authors touch on the differences in the way in which public’s views are accessed and I think that point bears further elaboration.**

Please see response to the point above regarding terms for patient data. We have added a sentence to the limitations about how the views expressed may have been affected by methods of studies. (Page 24)
